# Deubiquitylases and nucleases in bacterial symbiont-induced cytoplasmic incompatibility

**DOI:** 10.1042/BST20253047

**Published:** 2025-10-22

**Authors:** Seun O. Oladipupo, Mark Hochstrasser

**Affiliations:** 1 Department of Entomology, The Ohio State University, Columbus, Ohio, 43210, USA; 2 Molecular Biophysics and Biochemistry, Yale University, New Haven, Connecticut, 06520, USA

**Keywords:** cytoplasmic incompatibility, *Wolbachia*, nuclease, deubiquitylase, toxin-antidote, host modification

## Abstract

In myriad arthropod species, maternally transmitted symbiotic bacteria spread through populations by manipulating host reproduction, most frequently by a mechanism called cytoplasmic incompatibility (CI). CI occurs when bacterially infected males fertilize uninfected females, typically causing paternal chromatin condensation and segregation defects and usually embryonic arrest in the first zygotic cell cycle. Embryos survive if the female is similarly infected, which promotes bacterial spread. The endosymbiont best known for CI is *Wolbachia*, now widely used against mosquitoes that vector viral diseases such as dengue fever. Although CI is induced by *Wolbachia* resident in testes, mature sperm carry no bacteria, indicating they alter sperm in a way that, following fertilization, interferes with embryogenesis. CI-inducing factors (Cifs) are expressed from syntenic *Wolbachia cifA-cifB* genes. CifB is required in the male germline to induce CI, while CifA expression in the host female is sufficient to rescue viability. Importantly, CifA suppresses lethality through its binding to CifB. Different CifB proteins have distinct CI-relevant enzymatic functions, in particular, deubiquitylase and nuclease activities. Consistent with these genetic data, CifB is packaged into sperm during spermiogenesis. While sperm morphological disruption has been observed in fruit flies carrying *cif* transgenes, a causal role in CI is unclear. Also not understood is how maternally provisioned CifA rescues embryo viability. Exciting new findings with diverse symbiotic bacteria reveal *cifA-cifB*-like operons on extrachromosomal plasmids. These results suggest far wider deployment of *Wolbachia*-related CI factors than previously thought and multiple mechanisms for lateral *cif* gene transfer.

## Introduction

**Figure 1:**
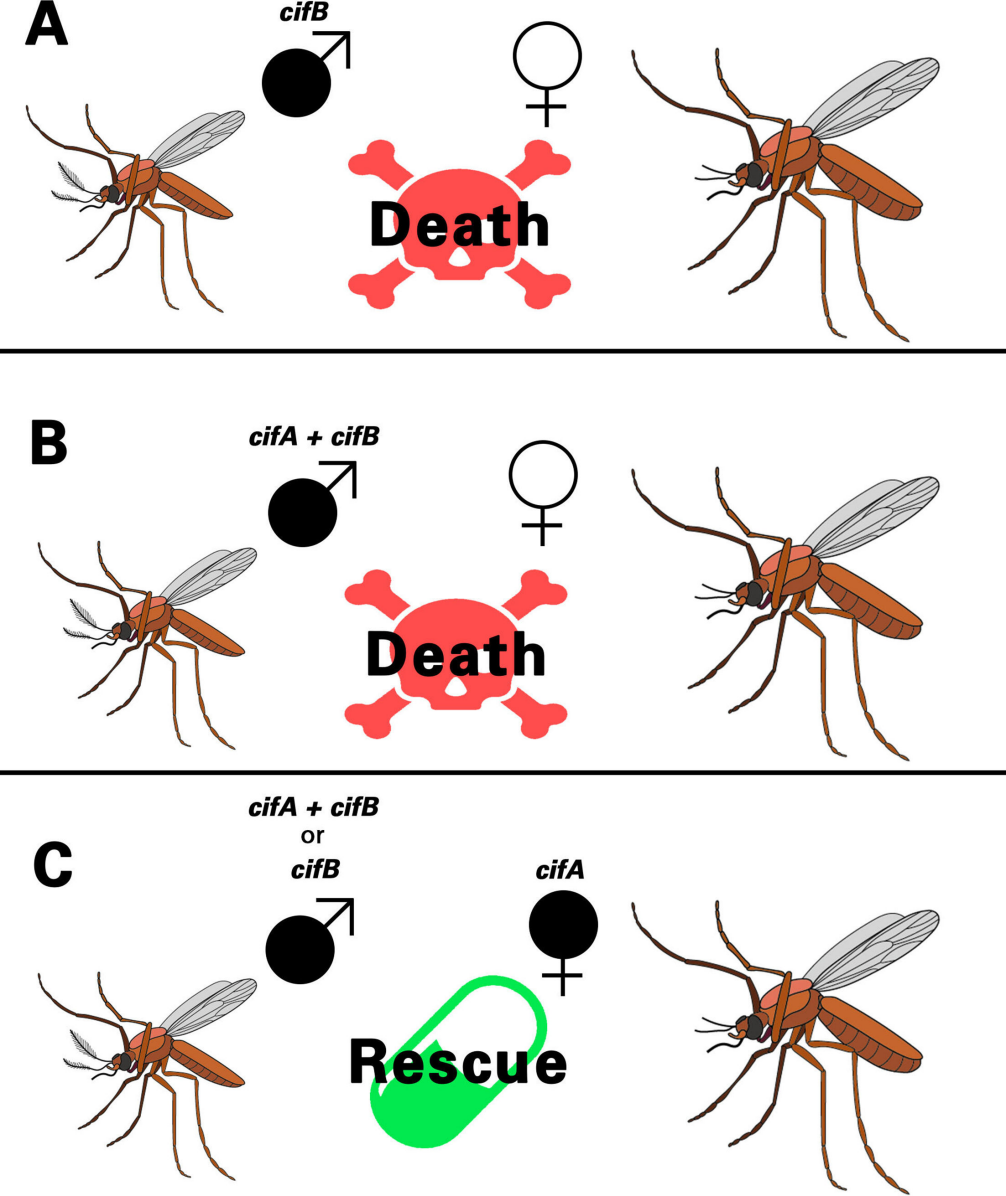
Cytoplasmic incompatibility and CI factors **A.** CifB expressed in the male germline is sufficient to induce embryonic death in some transgenic model systems when the males are crossed to uninfected females. **B.** In other transgenic models, expression of both CifA and CifB in males is required to induce CI in crosses with uninfected females. **C.** Rescue cross. When a *Wolbachia*-infected male is crossed with a female infected with the same *Wolbachia* strain, maternal CifA restores normal embryonic development. Filled-in symbols denote *Wolbachia* infection or *cif* transgene expression, while open symbols indicate uninfected or nontrangenic status.

Cytoplasmic incompatibility, or CI, is a type of conditional male sterility affecting a vast number of insect species and other arthropods [1–4]. CI is most commonly caused by infections with maternally inherited *Wolbachia*, which are endosymbiotic bacteria capable of several other reproductive manipulations as well [1,5]. In diploid species, CI usually leads to embryonic death when infected males mate with uninfected females; embryos most often arrest in the first zygotic cell cycle due to improper replication and segregation of the paternal genome, although arrest may occur at later stages [6–8]. When females are infected with the same *Wolbachia* strain as the male partner, embryo viability is rescued (Figure 1). Notably, in infected haplodiploid species, such as parasitoid *Nasonia* wasps, CI-induced elimination of the paternal genome usually leads to conversion of diploid embryos to haploid male embryos [1,5]. The bacteria are found in eggs [9], and they are present in the testes of CI-inducing males. However, they are absent from mature sperm [10]. Hence, the *Wolbachia* bacteria must trigger CI by altering the sperm in some way.

What has drawn interest in *Wolbachia*-mediated CI, besides the mysteries of the process itself, is its practical utility in insect and disease vector controls [11–15]. Because CI-inducing *Wolbachia* bacteria impose a conditional sterility on males, they have been used to deplete populations of arbovirus-carrying vectors such as the *Aedes aegypti* mosquitoes that transmit dengue fever, yellow fever, and Zika viruses [13,16]. Moreover, infection of insects by *Wolbachia* can greatly reduce their viral (or malaria-causing *Plasmodium*) loads [17–19]. CI is used to promote the spread of the bacteria into a naïve vector population and thereby suppress disease transmission even though insect levels themselves do not decrease [15].

The molecular factors responsible for CI remained unknown for many decades. The first step toward a solution was the description of a *Wolbachia* protein identified by mass spectrometry in dissected spermathecae – sperm storage organelles – from a laboratory population derived from uninfected female *Culex pipiens* mosquitoes fertilized by *Wolbachia*-infected males [20]. This protein is now called CifA*^w^* ^Pip^ or CidA*^w^* ^Pip^ (*‘w*Pip’” is one of many distinct *Wolbachia* strains). The authors noticed that the *cifA* gene was immediately upstream of another gene, *cifB*, in all CI-inducing *Wolbachia* genomes available at the time. These two CI factors (Cifs) were subsequently shown to be responsible for CI using experiments with transgenic *Drosophila melanogaster* [21,22]. In these and later studies with transgenic fruit flies and mosquitoes, expression of the *cifB* gene (and sometimes both *cifA* and *cifB*) was found to be required in the male germline to induce CI, while expression of *cifA* in females was sufficient to rescue embryo viability [21–28].

The nomenclature around Cifs reflects the evolving understanding of CI proteins. Cif serves as a general term for the proteins expressed from the two-gene operons responsible for CI [21,22]. The CifB proteins modify or are delivered through sperm and, following fertilization, lead to the developmental disruptions characteristic of CI. CifA proteins function as rescue factors when expressed in females, neutralizing the incoming cognate CifB in some way and restoring normal embryogenesis.

**Figure 2:**
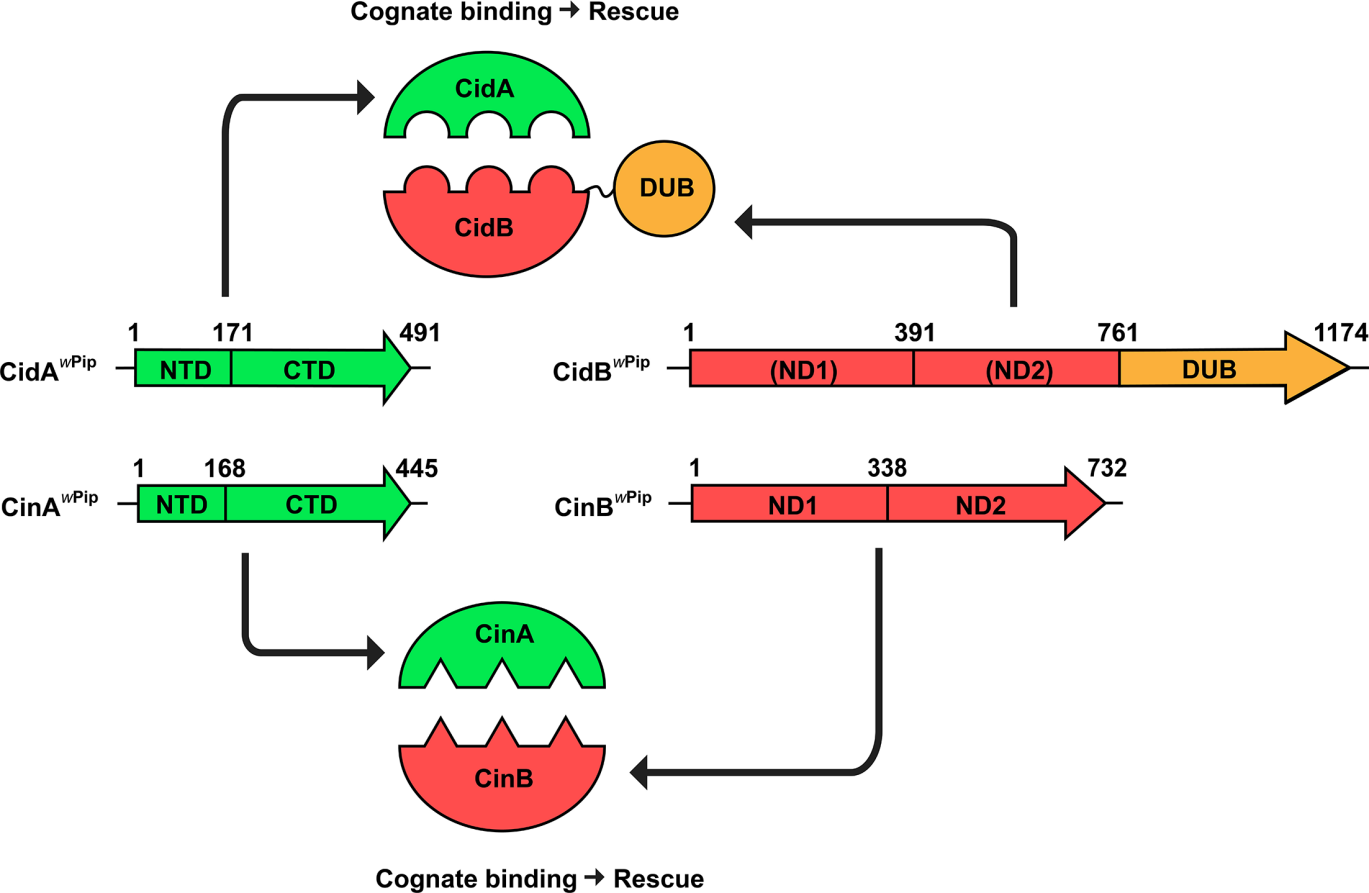
Operon-like structure of CI factor genes and cognate protein binding The *cidA-cidB* and the *cinA-cinB* operons depicted are from a *w*Pip *Wolbachia* strain identified in *Culex pipiens* mosquitoes. Cifs form cognate-specific protein complexes with extensive interfaces [29,30]. NTD, N-terminal domain of CifA; CTD, C-terminal domain. The CifA CTD consists primarily of six HEAT repeats. ND1 and ND2 refer to upstream and downstream nuclease domains in CifB proteins; key catalytic residues are missing in these domains in the CidB deubiquitylase (hence the parentheses). Two additional, more distantly related pseudonuclease-fold domains in both the CinB and CidB proteins are omitted here for clarity.

Further classification is based on (predicted) enzymatic domains in the CifB proteins. Initial phylogenetic analyses of the *Wolbachia* CI factors had sorted them into five evolutionary clades, with the encoded CifB proteins harboring different predicted enzymatic activities (Figure 2) [31]. The type I CifB has a C-terminal *d*eubiquitylase (DUB) domain and is named CidB (for CI-inducing DUB). Types II-IV have (predicted) active *n*uclease domains and are part of the CinB group. Finally, most type V CifBs are predicted to have both nuclease and DUB activities; these are therefore called CndB proteins and often have additional domains (not shown in Figure 2) [21,32].

**Figure 3:**
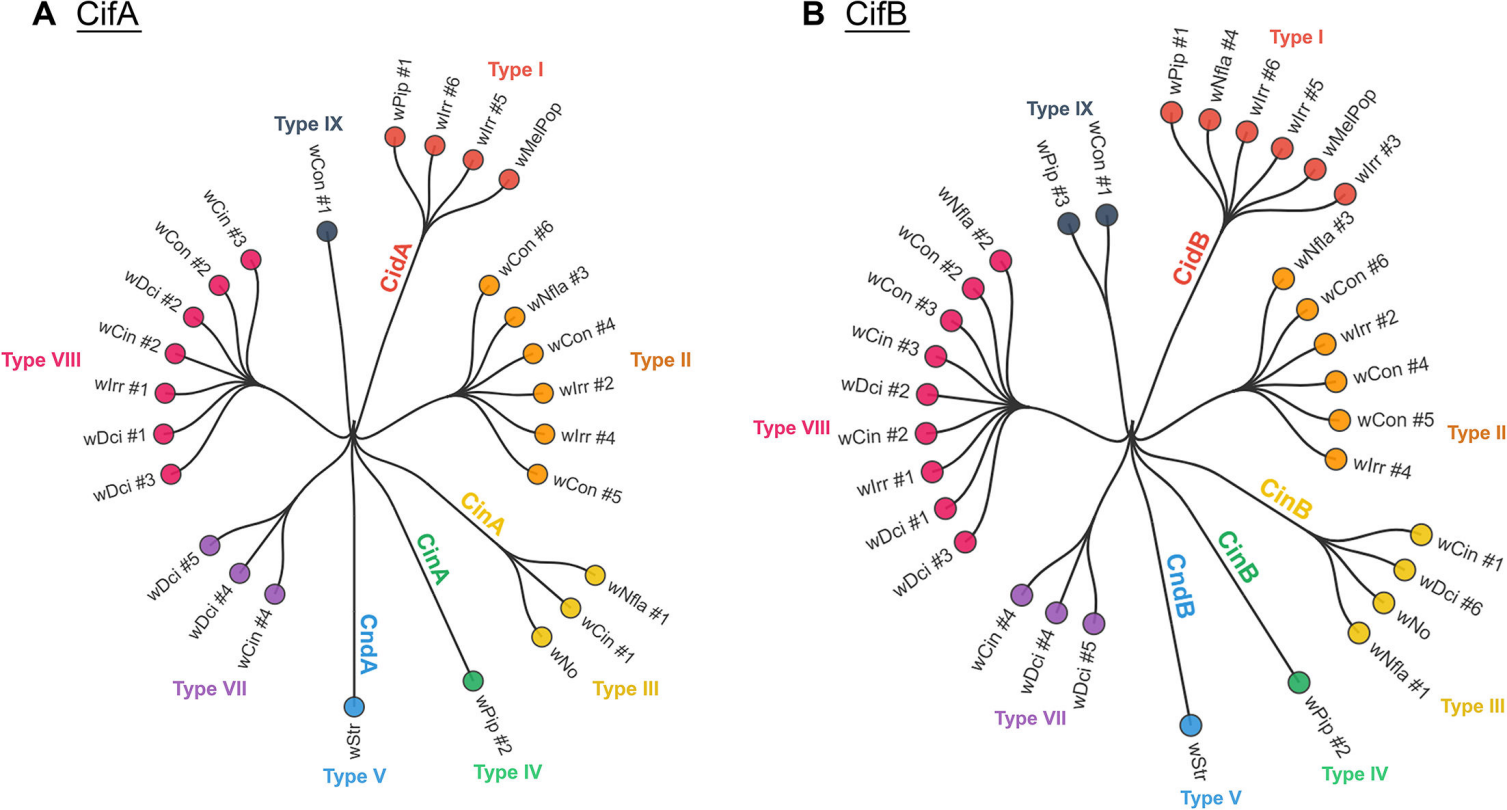
Evolutionary relationships among representative *Wolbachia* Cifs from major evolutionary clades. These unrooted phylogenetic trees display relationships between selected reference strains (i.e. bold strains from Tan et al. [33,34], [33] Figure 2 and Beckmann et al. [34] Figure 3 that represent major Cif types. Each strain designation includes the *Wolbachia* strain number (e.g. *w*Pip #2 indicates *Wolbachia* from strain *w*Pip, isolate #2). Each isolate is colored differently.

Remarkably, five additional *cifA-cifB* clades, mostly in *Wolbachia*, were recently identified in a multipronged bioinformatic study [33] (Figure 3). As is true of type V Cifs, the type VI-IX Cifs often involve large CifB proteins with additional domains. Of the ten CifB types, all except type I (CidB) are predicted to have active nuclease domains. Interestingly, some *Wolbachia* strains harbor multiple *cifA-cifB* operons from different clades. For example, the *w*Ltri strain that infects the leafminer *Liriomyza trifolii* and causes potent CI has three different Cif types (I, III, and V), suggesting the acquisition of *cif* genes through lateral gene transfer [35].

Understanding the exact functions and mechanisms of the CI factors would strengthen *Wolbachia*-based strategies aimed at mosquito population suppression and disease mitigation. For example, *Wolbachia* expressing either Cid or Cin protein pairs has been shown to be effective in suppressing *A. aegypti*-vectored transmission of dengue fever [15,28]. Transgenic expression of these proteins should allow fine-tuning of these strategies, including improving resistance to host mutations that might lead to reduced CI penetrance and bypassing potential negative effects of *Wolbachia* bacteria on fecundity and lifespan [29]

## Biochemical activities of *Wolbachia* CI factors

As noted above, sequence analyses of the CI proteins suggested potential enzymatic activities (i.e. nuclease or DUB) in specific domains of the CifB proteins [20,24]. The CifA proteins, by contrast, lack clear homology to known enzymes. When expressed in budding yeast, CifB proteins impair growth, whereas CifA expression is innocuous [21]. However, if the cognate pair of proteins (i.e. CifA and CifB from the same bacterial operon) are co-expressed in yeast, growth is again normal. These data indicate that the CifB proteins act as toxins and CifA factors as cognate-specific antidotes in yeast [21,24]. Such a toxin-antidote (TA) mechanism may also underlie CI.

CifA and CifB bind tightly to one another; for example, the CinA-CinB heterodimer from *w*Pip *Wolbachia* has a dissociation constant of 25 nM [24], and high-resolution crystal structures revealed an expansive interface (~2500 Å^2^ buried surface) between cognate pairs [30,36]. By mutagenizing residues at this interface, it was possible to show that CifA-CifB binding is required not only to suppress CifB toxicity in yeast but also to rescue embryo viability in transgenic CI crosses. Therefore, the requirement for binding between maternally provisioned CifA and paternally delivered CifB must be part of any mechanistic model for CI.

Interestingly, binding of CifA to CifB (both nuclease and DUB types) appears to stabilize the latter protein, which showed increased abundance when CifA was co-expressed; this was observed both in yeast [27,36] and in cultured *Drosophila* cells and is likely true during spermatogenesis as well [37]. Transgenic expression of CifA along with CifB in the testes is required for strong CI induction in some but not all cases; for example, coexpression of CidA*^w^* ^Mel^ or CinA*^w^* ^Pip^ with their cognate CifB is necessary in *D. melanogaster,* but testes coexpression of CinA*^w^* ^No^ with CinB*^w^* ^No^ is not [24,25,27]. Inhibition of CifB degradation by CifA binding may promote the loading of sufficient CifB into sperm nuclei for CI induction, and this requirement is likely sensitive to CifB expression levels.

The type I CifB proteins (Figure 3), such as those from the *w*Pip and *w*Mel *Wolbachia* strains, CidB*^w^* ^Pip^ and CidB*^w^* ^Mel^, respectively, have a domain with similarity to the Ulp1-like cysteine proteases (CE clan) [38] and were found to be DUBs, enzymes that cleave ubiquitin from ubiquitylated substrates (Figure 2). Using *D. melanogaster* transgenic for the *w*Pip *cidA-cidB* operon, DUB catalytic activity was shown to be necessary for CI induction by males [21,39]. However, expression of catalytically inactive CidB*^w^* ^Pip^ at higher levels still led to a CI-like phenotype, possibly due to interference with substrate function by persistent binding to the inactive enzyme [37,39]. Although CI-relevant substrates, such as importin-α and histone–protamine exchange factors, have been suggested, their exact mechanistic links to CI have not yet been established [40,41]. Alternatively, the DUB activity might protect CidB from ubiquitylation and degradation during spermiogenesis, as suggested by its reduced levels in post-meiotic spermatids when the DUB domain is delet [37]. The CI toxicity of CidB factors, in this view, may result from their binding to specific host chromatin targets rather than removing ubiquitin from them. Because of the inability to manipulate *Wolbachia* genetically, it has not yet been possible to determine whether loss of DUB catalytic activity in a normal infection context would interfere with CI and, if so, at what stage(s).

The CinB proteins are closely related in structure to the N-terminal regions of the CidB proteins (Figure 2) [27,36].CinB proteins have two intact PD-(D/E)xK sequence elements, a catalytic motif present in many RNases and DNases [42,43]. In some of these nucleases, a QxxxY motif is found just downstream of the PD-(D/E)xK sequence and may enhance substrate binding or nuclease activity [43]. In the CinB clades and the CndB clade (Cif type V), the key catalytic residues are all present in both PD-(D/E)xK motifs, and these motifs are all closely followed by a QxxxY element [27]. Although the nuclease fold remains, no catalytically intact PD-(D/E)xK motifs appear to be present in the type I CidB (DUB-containing) factors, and a proximal QxxxY element is absent as well [27,33]. Surprisingly, structural analysis revealed two additional, more divergent PD-(D/E)xK pseudonuclease domains in both the CinB and CidB factors, which also lack bound divalent cations [30,36]. Thus, all CifB proteins bear four (pseudo)nuclease domains [33].

Purified recombinant CinB*^w^* ^Pip^ (type IV CifB, see Figure 3) can cleave various DNA substrates at low rates and requires either Mn^+2^ or Mg^+2^ for activity [24,30]. While RNA cleavage was not observed, it is possible that this requires an additional cofactor or only occurs at specific sequences. Mutation of catalytic residues in either of the two major PD-(D/E)xK motifs abolishes DNase activity and also eliminates CinB*^w^* ^Pip^ toxicity in yeast without changing protein expression levels [24]. These data support the view that CinB*^w^* ^Pip^ is a nuclease and that this activity is critical for toxicity.

In a recent report, both CidA*^w^* ^Mel^ and CidB*^w^* ^Mel^ were also proposed to possess nuclease activities [34]. DNase and RNase activities against (deoxy)oligonucleotide substrates were attributed to CidA*^w^* ^Mel^, while CidB*^w^* ^Mel^ was reported to have DNase activity. This is unexpected based on the high-resolution structures available for these proteins [30,36]. The CidA*^w^* ^Mel^ and CidA*^w^* ^Pip^ structures revealed no known nuclease fold; instead, they contain a structurally unique N-terminal domain and a C-terminal domain with six HEAT repeats.

These recent enzyme activity claims should be treated with caution. The recombinant His_6_-tagged CidA*^w^* ^Mel^ and CidB*^w^* ^Mel^ proteins used by Kaur et al. [34]. were isolated using single-step affinity purification from *E. coli*. Mass spectrometry identified peptides from several *E. coli* RNases in the CidA preparation used for enzyme analysis. For the CidB*^w^* ^Mel^ analysis, it is more difficult to rule out a DNase activity because of the low conservation of PD-(D/E)xK motifs; however, the PD-(D/E)xK motifs that are found in CidB lack multiple key catalytic residues, and divalent cations were not observed in the crystal structures [21,30,33,36]. The CidB*^w^* ^Mel^ QxxxY sequence suggested as being relevant here is not conserved among CidB proteins from different CI-inducing *Wolbachia* strains such as CidB*^w^* ^Pip^, and mutating the QxxxY element to AxxxA in CidB*^w^* ^Mel^, while accompanied by loss of apparent DNase activity, appeared to cause misfolding since only ~40% of the mutant protein was intact, full-length protein [34]. Hence, while the CidA and CidB (class I Cif, see Figure 2) proteins may bind nucleic acids [24], their intrinsic ability to cleave them remains to be verified.

## Cytological manifestations of CI

**Figure 4:**
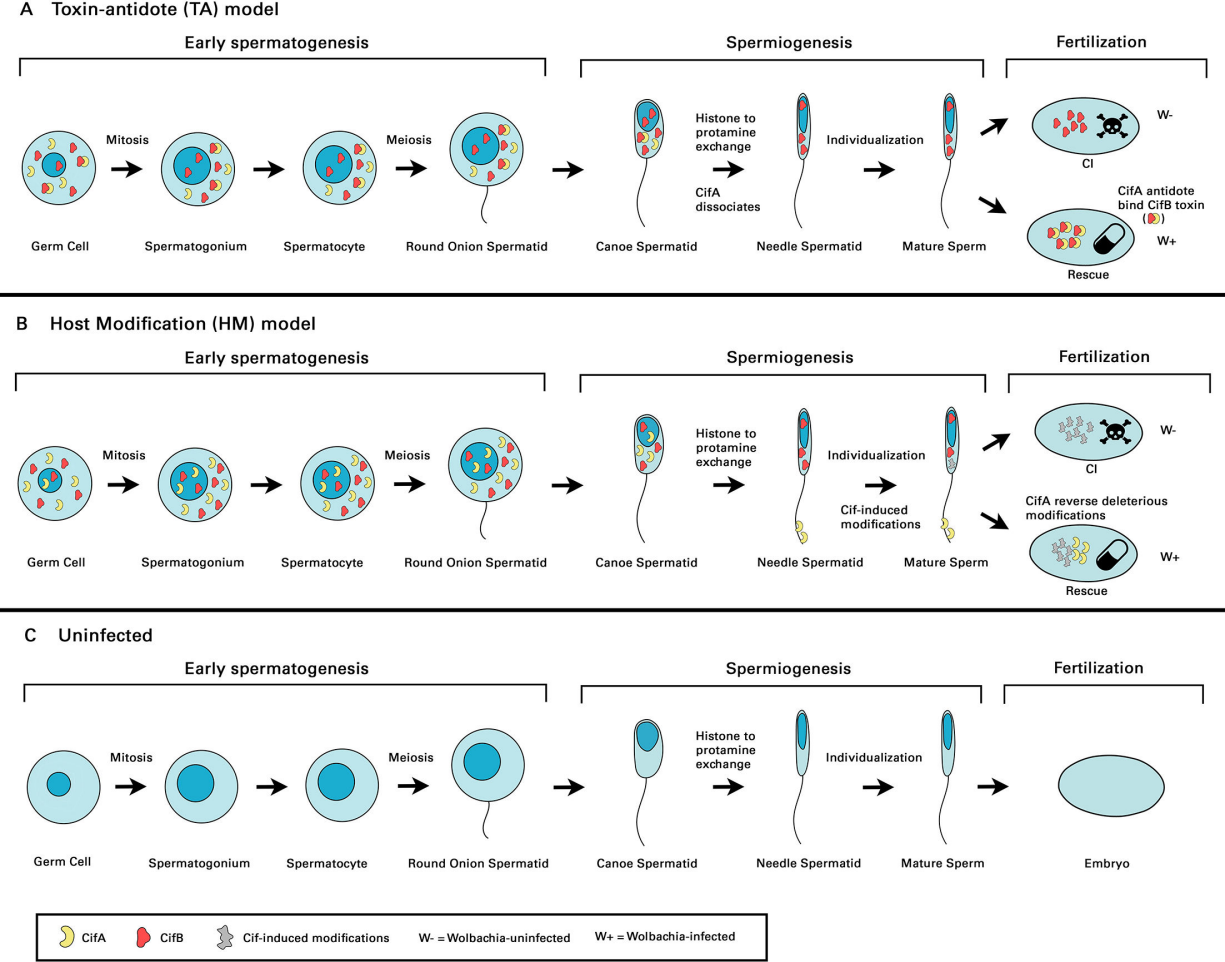
Hypothetical mechanisms for *Wolbachia*-induced CI. **A.** Toxin-antidote (TA) model: in *Wolbachia*-infected testes, CifA (antidote) and CifB (toxin) proteins appear to heterodimerize and persist through spermatogenesis at least until the histone-to-protamine transition. In some studies, CifA disappears after this point, leaving CifB to be loaded into mature sperm nuclei. Following fertilization of an uninfected egg (W-), CifB in the male pronucleus interferes with paternal chromatin replication and the ensuing chromosome condensation and separation during mitosis, arresting embryonic development. Conversely, fertilization of a *Wolbachia*-infected egg (W+), allows maternal CifA to bind to and neutralize CifB in the male pronucleus, thereby rescuing embryogenesis. **B.** Host-modification (HM) model: Cifs induce modifications during spermatogenesis that persist in mature sperm. Cif-induced modifications lead to persistent paternal chromatin defects following fertilization of an uninfected egg (W-). However, the presence of CifA in a *Wolbachia*-infected egg rescues embryogenesis by reversing the Cif-induced modifications or slowing maternal chromatin condensation and separation to synchronize with the altered male pronucleus (‘timing model’ [44]. In this model, CifA and CifB need not be transferred from sperm to egg, nor do they need to bind one another following fertilization. **C.** Uninfected hosts: spermatogenesis and fertilization in the absence of *Wolbachia* infection. Germline stem cells undergo mitosis to form spermatogonia, which divide and differentiate into spermatocytes. These cells complete meiosis to produce round spermatids that elongate into canoe-shaped and then needle-shaped forms. Following meiosis, histone-to-protamine exchange occurs, followed by individualization, resulting in mature sperm.

Understanding CI requires knowledge of the normal fertilization process and how *Wolbachia* disrupts it (Figure 4C). Upon fertilization, the sperm-derived male pronucleus undergoes nuclear envelope breakdown and exchanges protamines for maternally supplied histones on its DNA. The male and female pronuclei then juxtapose and undergo DNA replication prior to the first zygotic mitosis. In *Drosophila* and other insects, the pronuclei do not fuse but remain separated, with each set of parental chromosomes then occupying one half of the metaphase spindle before segregating during anaphase and then commingling [45].

Observations in diverse genera, including *Drosophila*, *Nasonia*, and *Culex*, have revealed highly similar cytological disruptions in incompatible crosses. CI specifically disrupts events in the paternal pronucleus. In incompatible crosses (*Wolbachia*-infected males × uninfected females), paternal chromatin fails to condense properly during the first cell cycle, leading to delayed chromosome segregation and characteristic bridging of paternal DNA at anaphase [7,46–48]. These defects result in abnormal chromosome inheritance and usually embryonic arrest. Critically, when females carry compatible *Wolbachia* infections, these disruptions are prevented and normal development proceeds, demonstrating the rescue function central to CI [1,7].

## Cellular defects induced by CI

Multiple, temporally distinct mechanisms likely underlie *Wolbachia*-induced CI. The delayed replication of paternal chromosomes is now recognized as an early and central CI defect [37,38]. Horard et al. demonstrated that CidB associates with paternal DNA regions exhibiting DNA replication stress, providing evidence that CidB may interfere with paternal DNA replication in incompatible crosses [38]. This replication interference manifests as prolonged retention of PCNA in the male pronucleus during metaphase, suggesting progression into mitosis with incompletely replicated DNA [49]. The resulting replication stress could account for the extensive chromosome bridging observed during anaphase, as segregation of unreplicated chromosomes creates persistent DNA bridges [49].

Recent work by Terretaz et al. revealed a potential mechanism by which CidB disrupts replication [37]. When CidB acts without CidA neutralization, it localizes to host chromatin during replication, interfering with the completion of DNA replication. Notably, CidA appears to relocalize CidB on chromatin, possibly preventing the toxin from blocking S-phase completion [37]. As noted above, additional CI-induced defects include disrupted chromosome condensation and cell cycle progression abnormalities. Impaired deposition of maternally supplied histone H3.3 results in abnormal chromatin assembly [50]. Moreover, Warecki et al. revealed that CI induces temporally distinct defects: approximately one-third of CI embryos bypass first-division failures but exhibit chromosome segregation errors during the mid-blastula transition [8]. These late defects resemble those caused by DNA replication inhibitors, suggesting persistent replication stress effects that become amplified when DNA synthesis naturally slows during development.

Collectively, these findings imply that CI functions as a transgenerational TA system, triggering paternal chromosome dysfunction by CidB disruption of replication progression. Maternally expressed CidA rescues embryo viability by binding to and potentially restricting CidB access to replicating chromatin targets in the male pronucleus.

## Localization of CI factors

The exact mechanistic basis of CI remains uncertain, especially the role of Cifs during spermatogenesis and post-fertilization. Current explanations fall into either TA or host-modification (HM) models (Figure 4). In the TA model (Figure 4A), CifB loaded into mature sperm nuclei induces embryo arrest after the sperm fertilizes the egg, with rescue achieved through the physical interaction of maternally supplied CifA and the incoming CifB in the early embryo [5,51]. Conversely, the HM model argues that co-expression of CifA and CifB in sperm precursors leads to harmful (chromatin) modifications, while CifA expressed in females mitigates these harmful effects after fertilization in some way [25,52]. In the HM scheme, the CI factors need not be present in mature sperm, nor is heterodimerization of CifA and CifB required for rescue (Figure 4B). Consequently, investigating the localization of Cifs in reproductive tissues and gametes could provide critical insights into how they cause CI.

In the first such study, by Horard et al. [39], localization of Cif proteins in cultured *Drosophila* cells strongly suggested that CI follows a TA mechanism. When the CidA*^w^* ^Pip^ and CidB*^w^* ^Pip^ proteins were co-expressed, both were cytoplasmic during interphase and colocalized on chromosomes during mitosis. CidA by itself behaved similarly. However, if CidB was expressed alone, it went to the nucleus during interphase, and cells underwent apoptosis. In the giant polytene cells of larval salivary glands, both proteins also co-localized, although in this case, they were bound along the interphase chromosomes [39]. Presumably, toxicity occurs when free CidB can enter the nucleus. Later structure-function analysis supported the view that CidA binding to CidB directly prevents CidB nuclear import [37]. It has been suggested that CidB and CinB use classical importins for transport into the sperm nucleus [40,41].

Cid localization in ovaries and oocytes has not been as extensively characterized. Results with *A. aegypti* mosquitoes transinfected with *w*Mel suggest CidA*^w^* ^Mel^ is mostly nuclear in ovarian cysts, although no staining in the oocyte itself was detected [52]. CidB*^w^* ^Mel^ was also not detected in the oocyte, but antibody reactivity was observed in the cytoplasm surrounding the large nurse cell nuclei. Despite not being seen in the oocyte, a weak signal for maternal CidA*^w^* ^Mel^ was present around the chromatin of embryonic nuclei [52]. When the same antibodies were used to analyze ovaries and embryos of the natural *w*Mel host, *D. melanogaster*, anti-CidA*^w^* ^Mel^ staining instead revealed weak cytoplasmic staining in the nurse cells, and no staining of either CidA or CidB was observed in early embryos from rescue crosses [34]. By contrast, Horard et al. [39] detected CidB*^w^* ^Pip^ – but not CidA*^w^* ^Pip^ – in the male pronucleus in fly eggs fertilized by *cidA-cidB* transgenic males. Finally, experiments with *w*No *Wolbachia*, which encode only a Cin nuclease operon in which CinB is sufficient for CI induction and CinA for CI rescue [27], revealed CinA*^w^* ^No^ concentrating in late-stage oocytes (Oladipupo et al. in preparation). CinB*^w^* ^No^ also localizes in the oocyte, suggesting physical interaction between the two proteins could play a role in rescue in the egg.

These often contradictory results make it difficult to assess how the CI factors participate in CI rescue. The exact point at which maternal CifA encounters paternal CifB to initiate rescue is not known but is a key issue for distinguishing between the TA and HM models. Higher resolution studies with sensitive and specific antibodies and fluorescent tags will be needed to resolve these questions. No data showing CifA-CifB association during the early mitotic divisions of the developing embryo have been described. Future research should therefore aim to clarify the functions of CI factors during early embryonic development [8].

Much debate also surrounds the localization and impact of the Cifs during spermatogenesis. Spermatogenesis is a multi-step process beginning with a germline stem cell dividing to yield a primary spermatogonium (Figure 4). This cell then undergoes multiple mitotic divisions, yielding the primary spermatocytes. In the ensuing meiotic stage, the diploid primary spermatocytes undergo reductional division to form haploid spermatids. Finally, during spermiogenesis, chromosomal histones are exchanged for a set of small basic proteins called protamines, which along with other major morphological changes gives rise to mature sperm [53,54].

All available studies agree that the cognate Cif protein pairs, including both CidA and CidB from *w*Mel and *w*Pip and CinA and CinB from *w*No *Wolbachia*, concentrate in cell nuclei in the stages of spermatogenesis prior to histone–protamine exchange ([39,52,55] and Oladipupo et al. in preparation). In one study of transgenic CidA*^w^* ^Pip^ and CidB*^w^* ^Pip^ expression in the *Drosophila* testes, only CidB*^w^* ^Pip^ persists in spermatids after the histone–protamine transition and, as noted above, decorates paternal chromatin in the early embryo [39]. Similarly, CinB*^w^* ^No^ could still be detected in mature sperm in the seminal vesicles, whereas CinA*^w^* ^No^ only associates with spermatids before the histone–protamine exchange, as had been seen for CidA*^w^* ^Pip^ (Oladipupo et al. in preparation). This was true for both transgenic expression in *D. melanogaster* and natural *w*No infections of *D. simulans*.

By contrast, analysis of CidA and CidB of *w*Mel in *D. melanogaster* revealed nuclear association of both proteins throughout spermatogenesis, except CidA*^w^* ^Mel^ seemed to move to the sperm tails once the sperm had matured [55]. CidA*^w^* ^Mel^ and CidB*^w^* ^Mel^ expressed in *w*Mel-infected *A. aegypti* exhibit similar cytonuclear behaviors, with CidB*^w^* ^Mel^ also localizing to the head and CidA*^w^* ^Mel^ to the tail of mature sperm based on staining with the same antibodies used in the earlier *D. melanogaster* analysis [52]. The authors noted disruption of histone–protamine exchange in CI-inducing males in these two studies and considered this disruption of chromatin integrity as the likely basis for an HM mechanism of CI. It should be noted that others have documented extensive spermatid chromatin defects caused by *w*Ri infection of *D. simulans* sperm cysts but argued that the resulting defective sperm were unlikely to fertilize eggs and therefore could not account for CI [56]. Notably, fertilization occurs at similar frequencies in matched CI and non-CI crosses [8,57].

## CI caused by other bacterial symbionts

Besides *Wolbachia*, several other bacteria can induce CI in different arthropod species, and an obvious question is whether related CI factors are involved. Certain species from the genera *Cardinium* and *Spiroplasma* are known to cause CI, but their genomes lack apparent *Wolbachia*-related *cif* operons, suggesting an independent origin of CI in these microbes [58,59]. Other symbionts, such as *Rickettsiella*, a γ-proteobacterium, have been shown to induce CI, but their genome sequences have not yet been determined [49,60]. However, another α-proteobacterium, *Candidatus Mesenet longicola*, has a *cndA-cndB* locus that could be responsible for the CI observed in infected coconut beetles [61].

Several newer studies now suggest that syntenic genes related to those responsible for CI in *Wolbachia* will also be relevant to CI caused by more divergent bacterial symbionts. Owashi et al. [62] report that a *Rickettsia* species closely related to *Rickettsia bellii* causes robust CI in its hemipteran host, and DNA sequencing of the bacteria revealed a pair of loci with *cifA*-like and *cifB*-like genes. Notably, these genes are on two different bacterial plasmids and are closely related to the *cnd* (class V) genes found in a plasmid in *Rickettsia felis*, whose booklouse host undergoes parthenogenesis [63]. Plasmids with *cif*-related genes have also been reported in some *Wolbachia* strains [64].

Perhaps even more provocative is the recent discovery of a widespread but previously overlooked endosymbiont, *Symbiodolus*, which infects members of at least six different insect orders [65]. As with *Wolbachia*, it is found in high numbers in multiple tissues, especially in ovaries, consistent with transovarial vertical transmission. While CI was not directly tested, other types of reproductive manipulation alter sex ratios of progeny, and this was not observed. Thus, a CI-like mechanism remains as a potential explanation for the high bacterial penetration into infected populations. *Symbiodolus* genome sequencing from 16 different host species revealed high overall conservation. The genomes included several plasmids. Remarkably, although not noted by the authors, *cifA-cifB* gene pairs are present in these plasmids. It is therefore tempting to hypothesize that these genes function as in *Wolbachia* to induce CI and promote *Symbiodolus* spread in host populations. As suggested by others [33,63,64], the presence of CI genes on plasmids, often near transposon sequences, suggests multiple means of lateral gene transfer of CI factors. CI functionality in these various examples remains to be tested by transgenesis experiments, but it appears that the CI factors first discovered in *Wolbachia* might be utilized far more widely than previously suspected in CI and potentially other reproductive manipulations.

## Outlook

CI induced by symbiotic bacteria is a fascinating example of a genetic drive mechanism that promotes bacterial inheritance in infected hosts. There are likely several distinct molecular mechanisms for CI caused by different microbes, as is true for male killing, another means by which intracellular bacteria promote their vertical transmission through the female germline. Nonetheless, as more examples are uncovered, it has become apparent that the CI factors first described in *Wolbachia* are likely to be deployed not only in related *Rickettsiales* bacteria but also in other far more distantly related microbes, such as the *Symbiodolus* γ-proteobacteria. Rigorous biochemical, genetic, and cell biological studies will be needed to paint a full mechanistic picture of CI from these multifarious examples. This knowledge will be essential for optimizing the use of CI in the field for insect pest and disease-vector controls.

**Perspectives**

- *Wolbachia*-induced cytoplasmic incompatibility (CI) is central to worldwide insect biocontrol efforts. Understanding its mechanisms will maximize its utility in these applications.
- Two current general explanations for CI that are not yet resolved are the toxin-antidote (TA) and host-modification (HM) models.
- Additional cell biological and biochemical studies of the CI factors should help distinguish between TA and HM mechanisms and might show these mechanisms are not as distinct as presently thought.

## Abbreviations

CI: cytoplasmic incompatibility
Cif: cytoplasmic incompatibility factor
DUB: deubiquitylase
HM: host modification
TA: toxin-antidote
